# Self-control during childhood and mental health at age 15–16: a longitudinal population-based study

**DOI:** 10.1007/s00787-025-02898-0

**Published:** 2025-10-29

**Authors:** Susanne R. de Rooij, Lisa van Dijke-Dieleman, Dominic Weinberg, Tanja G. M. Vrijkotte

**Affiliations:** 1 Department of Epidemiology and Data Science, Amsterdam UMC, University of Amsterdam, Meibergdreef 9, Amsterdam, 1105 AZ the Netherlands; 2 Amsterdam Public Health Research Institute, Amsterdam, the Netherlands; 3 Amsterdam Reproduction and Development, Amsterdam, Netherlands; 4 Department of Public and Occupational Health, Amsterdam UMC, University of Amsterdam, Amsterdam, the Netherlands; 5 Clinical Child and Family Studies, Erasmus School of Social and Behavioural Sciences, Erasmus University Rotterdam, Rotterdam, The Netherlands

**Keywords:** Self-control, Conduct problems, Childhood, Adolescence, Mental health, Gender differences

## Abstract

Low self-control in adults is associated with a broad range of negative outcomes. However, studies on the long-term impact of low self-control on mental health outcomes in youth are limited. We examined associations between low self-control during childhood (age 5–12) and mental health in adolescence (age 15–16), with specific interest for gender differences and whether conduct problems acts as a mediator. Data from a longitudinal birth cohort (Amsterdam Born Children and their Development) study was used (*n* = 1775). We scored self-control using items from the Strengths and Difficulties Questionnaire, Behavior Rating Inventory of Executive Function and Substance Use Risk Profile Scale collected at ages 5–6 and 11–12 years by multiple raters. Self-esteem (Rosenberg Self-esteem Scale), subjective well-being (Cantril-ladder), depression/anxiety symptoms (Youth Self Report) and perceived stress (Perceived Stress Scale) were mental health outcomes at age 15–16. We stratified by gender and tested mediation by conduct problem, as measured by the Strengths and Difficulties Questionnaire. Higher self-control was associated with higher self-esteem, higher subjective well-being, fewer depression/anxiety symptoms and less perceived stress in boys and girls (*p* < 0.01). Overall, boys had lower self-control during childhood. Associations between self-control and mental health outcomes were stronger in girls than in boys. Conduct problems functioned as a (partial) mediator, but only in girls. Children with higher self-control had better mental health during adolescence, especially girls. In girls, conduct problems (partially) explained this association. Research is needed to find out if interventions, directed at enhancing self-control in childhood, are beneficial for later mental health.

**Supplementary Information:**

The online version contains supplementary material available at 10.1007/s00787-025-02898-0.

## Introduction

Mental health problems typically emerge for the first time during adolescence [1, 2]. Currently, approximately 20% of adolescents globally have some form of a mental health issue [3]. There are clear gender differences in mental health during adolescence. Generally, girls have more mental health problems than boys [4]. There are also differences in the type of mental health problems, as girls more often show symptoms of anxiety and depression, struggle more often with eating disorders and have more perceived stress, whereas boys generally show more substance abuse and behavioral problems [5, 6]. Mental health problems during adolescence are a huge societal problem as they are associated with subsequent worse general health, more substance abuse, lower academic achievements, more delinquent behavior and more unemployment [7]. Factors from childhood could significantly influence the emergence of mental health issues during adolescence, and one such factor might be self-control. Childhood would then present a potential opportunity to enhance self-control abilities through preventive and intervention measures, thereby mitigating subsequent adverse mental health outcomes.

Self-control is an individual’s ability to regulate emotions, thoughts and actions. It is best described as an umbrella term that includes the regulation of impulses, self-discipline, control of attention, the regulation of emotions and perseverance [8]. Self-control enables an individual to work towards long-term rewards in the face of conflicting urges that give immediate gratification [9]. Low self-control in adults has been associated with a broad spectrum of negative outcomes, including more behavioral problems, higher rates of criminal behavior, less social support, higher levels of substance abuse, lower academic achievements and even a shorter lifespan [10–12].

Children generally demonstrate lower levels of self-control compared to adults, attributable to the ongoing development of their cognitive and emotional regulatory capacities. However, there is considerable variability among children [13]. There are also sex differences with girls generally having more self-control than boys [14]. Low self-control skills during childhood can have negative repercussions on the long term. A landmark study by Moffitt et al. in 2011 showed that New Zealand children with low self-control repeatedly measured between 3 and 11 years of age had more health problems, financial problems, substance dependence and higher levels of criminal conviction records at an adult age [8]. These findings have since then been replicated in studies conducted in the US [15, 16]. Another study from New Zealand showed comparable associations between self-control repeatedly measured during ages 6–12 years and outcomes at adult age [17]. The associations in this study seemed to be largely explained by conduct problems during childhood (6–10 years of age) [17]. The authors hypothesized that low self-control could be a precursor for childhood conduct problems or that it is a symptom of broader externalizing problems in children. Other studies have shown associations between lower self-control and more conduct problems in adolescents as well as between childhood conduct problems and later mental health problems [18–21]. Conduct problems may thus mediate the association between childhood self-control and mental health in later life.

A few studies investigated a potential role of childhood self-control in subsequent mental health issues. The two aforementioned studies from New Zealand did not find an association between self-control during childhood and depression as an adult, but one found an association with anxiety, which was no longer statistically significant after controlling for conduct problems [8, 17]. One other longitudinal study showed a positive association between self-control during childhood (repeatedly measured between ages 3–11 years) and life satisfaction as an adult [22]. Another study in adolescents demonstrated associations between self-control at age 11–14 and anxiety symptoms but also not depressive symptoms, with a follow-up period of 7 months [23]. Cross-sectional studies in young adults and adults have demonstrated links between low self-control and higher levels of perceived stress, lower self-esteem, and more internalizing problems such as symptoms of depression or anxiety [10, 24], but also that high self-control is associated with improved life satisfaction, more general happiness and higher subjective well-being [24–27]. Having high self-control could lead to more positive emotions which could improve mental health [25]. Alternatively, the positive association between self-control and mental health could be due to a higher likelihood to achieve goals, more focus on acquiring positive gains and less focus on avoiding losses [26]. However, these studies in (young) adults were all cross-sectional and do not rule out a bidirectional association between self-control and mental health.

The current study focused on the association between self-control during childhood with repeated measurements at ages 5/5 and 11/12 and subsequent adolescent mental health, gender differences in this association, and conduct problems as a potential mediator of this association. We hypothesized that low self-control during childhood would be associated with lower levels of self-esteem, lower subjective well-being, more perceived stress and more anxiety/depression symptoms at age 15–16 and that conduct problems would mediate these associations. Given gender differences in self-control and mental health, we also explored differences in these associations for boys and girls.

The study used data from the Amsterdam Born Children and their Development (ABCD) study, a longitudinal population-based cohort study of 8000 children born in Amsterdam, the Netherlands. In line with the studies by Moffitt et al. [8], Fergusson et al. [17], and Richmond-Rakerd et al. [22], we constructed a self-control item from several aspects of self-control measured at ages 5–6 and 11–12, subsequently relating this overall childhood self-control item to mental health outcomes measured at age 15–16.

## Method

### Study population

The Amsterdam Born Children and their Development (ABCD) study is a prospective population-based cohort study of 8000 children who were born in Amsterdam, the Netherlands [28]. Between January 2003 and March 2004, all pregnant women living in Amsterdam (*n* = 12,373, covering an estimated ≥ 99% of the target population) were invited to participate during their first prenatal visit to a general practitioner, midwife or gynecologist. A total of 8266 women (67%) returned a completed questionnaire that was sent to their homes (phase 1). Mothers and children were followed up during infancy (phase 2), at age 5–6 years (phase 3), at age 11–12 (phase 4) and age 15–16 (phase 5). The present study used data from phases 3, 4 and 5. We included children with enough data available to calculate a self-control score and with data available for at least one of the four mental health outcomes (see below). Of the 4582 children included in phase 3, 1998 children were excluded because they had incomplete information on self-control measures at age 5–6 or 11–12. Furthermore, 809 children were excluded because there was no outcome data available at age 15–16. In total, 1775 children were included in the study, of whom 829 (46.7%) were boys and 946 (53.3%) girls (Supplemental Fig. 1). Approval of the study was obtained from the Central Committee on Research involving Human Subjects in the Netherlands, the Medical Ethical Committees of participating hospitals, and from the Registration Committee of Amsterdam, and was conducted according to the Declaration of Helsinki. Each mother gave written informed consent for herself and her child, and the children themselves provided consent at age 16.

### Self-control

Our measure of self-control across childhood was based on the work of Moffitt et al. [8], Fergusson et al. [17], and Richmond-Rakerd et al. [22], as discussed in the Introduction, although not constructed in exactly the same way (fewer repeated measurements and using different scales). In all three studies, self-control was assessed through repeated measurements collected at various points during childhood (ages 3–11 and 6–12), using reports from multiple informants—including parents, teachers, and the children themselves—to capture different facets of the construct. We here used data collected at age 5–6 years and at age 11–12 years to construct the self-control measure during childhood (fewer repeated measurements compared to the other studies). From the data collected at age 5–6, we used the ‘hyperactivity/inattention domain’ (5 items) from the Strengths and Difficulties Questionnaire (SDQ) [29] which was filled in by the mother (Cronbach’s α = 0.75) and the school teacher (α = 0.84). From data collected at age 11–12, we used the same five items from the SDQ, which was filled in by the mother (α = 0.80), the school teacher (α = 0.86) and the child (α = 0.86). We also used nine items from the Behavior Rating Inventory of Executive Function (BRIEF) [30] filled in by the mother (α = 0.83), that focused on self-control in the domains of working memory, emotional control and inhibition. Finally, we used five items from the Substance Use Risk Profile Scale (SURPS) (α = 0.74) [31], which were all in the impulsivity domain. Supplemental Table 1 provides an overview of all the self-control items.

**Table 1:** Demographic characteristics and covariates per self-control quintile

	Self-control in quintiles					
	Q1 (lowest)	Q2	Q3	Q4	Q5	Total
Categorical variables						
N	355	355	355	355	355	1775
Gender						
Boys	224 (63.1)	198 (55.8)	157 (44.2)	147 (41.4)	103 (29.0)	829 (46.7)
Girls	131 (36.9)	157 (44.2)	198 (55.8)	208 (58.6)	252 (71.0)	946 (53.3)
Ethnicity of maternal origin						
Dutch	267 (75.2)	274 (77.2)	260 (73.2)	287 (80.8)	282 (79.4)	1370 (77.2)
Other western	41 (11.5)	43 (12.1)	58 (16.3)	36 (10.1)	36 (10.1)	214 (12.1)
Non-western	47 (13.2)	38 (10.7)	37(10.4)	32 (9.0)	37 (10.4)	191 (10.8)
Household						
Two-parent household	279 (78.6)	303 (85.4)	309 (87.0)	310 (87.3)	308 (86.8)	1509 (85.0)
Single-parent household	42 (11.8)	25 (7.0)	17 (4.8)	18 (25.0)	25 (7.0)	127 (7.2)
Other	33 (9.3)	20 (5.6)	24 (6.8)	25 (7.0)	10 (2.8)	112 (6.3)
SES parents						
ISCED 1	19 (5.4)	6 (1.7)	8 (2.3)	1 (0.3)	10 (2.8)	44 (2.5)
ISCED 2	68 (19.2)	36 (10.1)	37 (10.4)	21 (5.9)	29 (8.2)	191 (10.8)
ISCED 3	265 (74.6)	305 (85.9)	302 (85.1)	325 (91.5)	306 (86.2)	1503 (84.7)
Age child at age 15–16 data collection (years)	15.9 (0.4)	15.9 (0.4)	15.9 (0.4)	15.9 (0.4)	15.9 (0.4)	15.9 (0.4)
Age mother at age 11–12 data collection (years)	44.4 (4.5)	45.3 (3.9)	45.3 (4.0)	45.3 (3.6)	44.8 (3.7)	45.0 (4.0)
Age father at age 11–12 data collection (years)	47.4 (6.0)	46.8 (5.0)	47.3 (5.1)	47.3 (5.1)	46.9 (5.7)	47.1 (5.4)
IQ child (Raven’s score)	14.4 (6.3)	15.7 (5.8)	16.4 (5.4)	16.6 (5.6)	17.2 (5.7)	16.1 (5.8)
SDQ conduct problems (score)	1.5 (0.9)	1.0 (0.6)	0.7 (0.6)	0.6 (0.5)	0.4 (0.4)	0.8 (0.7)

Data are given as numbers (%) and means (SD), *SES* socioeconomic status, *ISCED* international standard classification of education, *SDQ* strengths and difficulties questionnaire

In these measures, a higher score indicated lower self-control. To this end, we had to invert some of the data. To calculate an overall child self-control score, each child’s total score per questionnaire was standardized (Z-score). These scores were then summed and divided by the number of questionnaires filled in per child (maximum of 7) to get an overall self-control score per child. The final self-control score was therefore a continuous variable.

To assess whether our construct indeed reflects a single self-control factor, we conducted a confirmatory factor analysis (CFA) using the lavaan package in R on the seven scales upon which the measure was based. We used the WLSMV estimator for categorical data and accounting for non-normality of distributions. Fit indices indicated an acceptable to good model fit (CFI = 0.98, TLI = 0.97, RMSEA = 0.08, SRMR = 0.06). Factor loadings ranged from 0.49 to 0.90. These outcomes suggest that the one-factor solution provides a moderate to good fit to the data.

For our descriptive table and for the graphical presentation of the results, the self-control variable was categorized into quintiles. In addition, we calculated separate self-control scores for age 5–6 and age 11–12 to evaluate the development and relative stability of self-control. We calculated these scores in the same way as our overall self-control score. We examined the correlations between the individual questionnaires, self-control per age period and overall self-control.

### Outcome measures

As outcome measures at the age of 15–16 years, we focused on the mental health factors self-esteem, subjective well-being, depression/anxiety symptoms and perceived stress. The Rosenberg Self-Esteem Scale (RSES) was used to measure self-esteem [32]. We used a short version containing five items (*n* = 1505), of which the reliability was α = 0.82. The Cantril ladder was used to score subjective well-being with the question ‘Rate your life on a scale from 1–10’ (*n* = 1683) [33]. The Youth Self Report (SR) focuses on anxiety and depressive symptoms [34]. We used a short version of 16 questions (*n* = 1773), with α = 0.87. Finally, the 10-item Perceived Stress Scale (PSS) was used to measure perceived stress (*n* = 1434), with α = 0.78 [35].

### Demographic characteristics, covariates and mediator

To describe our study population, we included the variables gender, ethnicity of maternal origin (Dutch, other western and non-western, based on the country of birth of the mother and grandmother of the participants), household characteristics (two-parent household, single-parent or other), mean age of the child during the age 15–16 data collection, age of the mother during the age 11–12 data collection, and age of the father during the age 11–12 data collection. As a covariate, we included socioeconomic status (SES) because the literature shows that higher self-control is associated with higher SES of the parents [8, 17]. SES was measured using the highest level of education from either parent assessed during the age 11–12 data collection and applying the three categories based on the International Standard Classification of Education (ISCED) (category 1 = low education (no education, early childhood, pre-primary, primary, lower secondary stage or second stage of basic education (< 6 years education)), category 2 = medium education (upper secondary, post-secondary, non-tertiary (6–9 years education)) and category 3 = high education (short-cycle tertiary, Bachelor, Masters, Doctoral or equivalent (≥ 10 years education)) [36]. Conduct problems was investigated as a mediator and determined by the Conduct problems domain from the SDQ measured during the age 11–12 data collection, which was filled in by the mother (α = 0.53), teacher (α = 0.61) and the child (α = 0.40). These scores were added together and divided by the number of questionnaires filled in.

### Statistical analysis

A non-response analysis was performed to examine the representativeness of the included children. The included children were compared to the excluded children with regard to: ethnicity of maternal origin, age of the mother, age of the father, household characteristics (all during the age 5–6 data collection), IQ, gender, socioeconomic status of the parents and conduct problems by T-tests (continuous variables) and Chi-square tests (categorical variables).

To examine development and stability of self-control over time, we made a table and Sankey diagram with self-control in quintiles during the age 5–6 and age 11–12 data collection phases. We applied Spearman’s rank correlation coefficients to quantify the stability of self-control over time.

We stratified all analyses by gender applying concurrent, side-by-side models, because the literature shows differences in self-control by gender and differences in mental health outcomes by gender [5, 6, 14]. We also formally tested whether gender was a significant effect-modifier for each outcome variable by adding an interaction term.

We applied linear regression analysis to assess the association between the self-control measure and the four mental health outcomes (model 1) and additionally controlling for SES (ISCED) (model 2). Mediation analysis was performed using the PROCESS tool for SPSS [37]. We assessed the effect of self-control on conduct problems (the *a* path), the effect of conduct problems on mental health outcomes (the *b* path), the simple effect of self-control on mental health outcomes (*total* effect or *c* path), the effect of self-control on mental health outcomes controlling for conduct problems (the *direct* or *c*′ path), and the effect of self-control through mediator conduct problems on mental health outcomes (the *indirect* effect as the product of *a* and *b*). A 95 percentile bootstrap CI was calculated based on 5,000 bootstrap resamples for the indirect effect, in order to test for significance. See Supplemental Fig. 2 for a schematic overview of this analysis.

Furthermore, we performed a sensitivity analysis on a subgroup of children with available IQ scores at age 11–12 years. Previous longitudinal studies have shown that higher self-control is associated with a higher IQ and it is therefore important to control for IQ of the child [8, 17]. We measured the IQ of the child using the Raven’s intelligence test [38]. The models for the sensitivity analysis are the same as models 1 and 2, but with additional adjustment for IQ in model 2, and will be referred to as models 1a and 2a.

P-values of less than 0.05 were considered to be statistically significant.

## Results

### Non-response analysis

In total, we excluded 2807 children. Compared to the excluded group, included children were more often of Dutch ethnicity of maternal origin (77.2% versus 57.0%, *p* < 0.001) and more often growing up in a two-parent household (90.2% versus 79.8%, *p* < 0.001). The included children also showed a lower level of conduct problems (M = 0.8 versus M = 1.1, *p* < 0.001). Parents of the included children were more highly educated (ISCED 3 77.3% versus 52.5%, *p* < 0.001) and both the fathers and the mothers were a little bit older (M = 40.6 versus M = 39.9, *p* < 0.001 for the fathers and M = 38.1 versus M = 36.8, *p* < 0.001 for the mothers). See Supplemental Table 2 for a complete overview of the non-response analysis.

### Characteristics of the study sample

There were more girls in the higher quintiles of self-control (and more boys in the lower quintiles). The children in the higher quintiles of self-control scored higher on the IQ test and had fewer conduct problems. The other characteristics did not show notable differences across the different quintiles (Table 1). Supplemental Table 3 shows summary statistics of the different conduct problem and self-control scales per age group and per gender.

### Self-control development and stability

Self-control in quintiles at age 5–6 and at age 11–12 were compared. A total of 565 children (31.8%) stayed in their quintile, meaning that 1210 (68.2%) of the children moved to another quintile: 642 children (36.2%) moved 1 quintile, 362 children (20.4%) moved 2 quintiles, 168 children (9.5%) moved 3 quintiles. A total of 18 children (1.0%) moved from the highest quintile to the lowest and 20 children (1.1%) moved from the lowest quintile to the highest. We calculated a Spearman’s rank correlation coefficient of 0.42 [95% CI 0.38, 0.46], which indicates weak to moderate correlation between self-control at age 5–6 and at age 11–12. Supplemental Fig. 3 shows a graphical representation of the stability of self-control across the quintiles and Supplemental Table 4 shows a full overview of the classification of children in quintiles at age 5–6 and at age 11–12.

### Gender differences in self-control, mental health and conduct problems

**Fig. 1:**
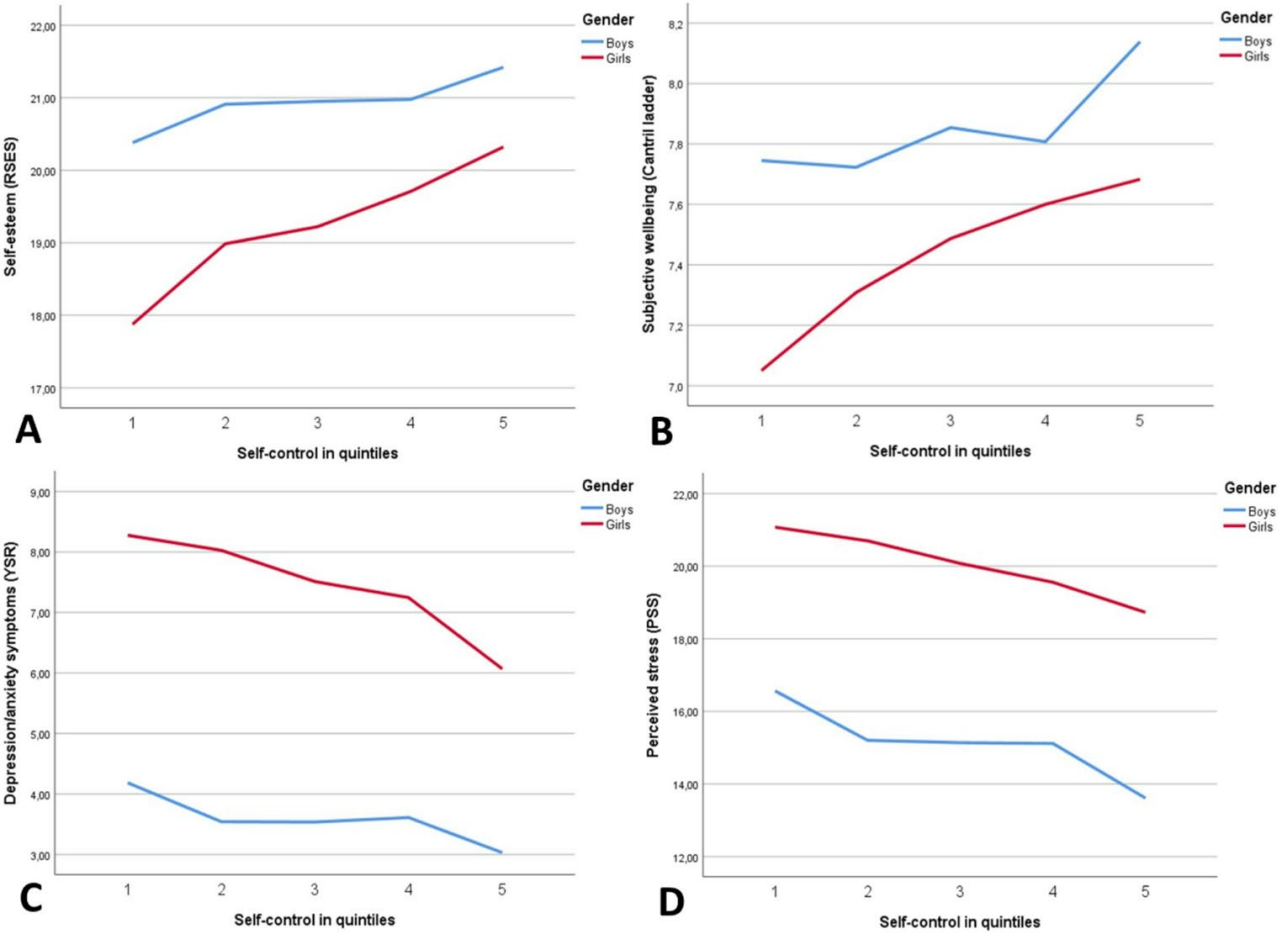
Association between self-control in quintiles and self-esteem (**A**), subjective well-being (**B**), depression/anxiety symptoms (**C**) and perceived stress (**D**), stratified by gender

A correlation table stratified per gender showed that the different questionnaires of conduct problems, self-control, self-control per age group and overall self-control were positively and significantly correlated in both genders (Supplemental Table 5). Overall girls had higher self-control than boys (Z-score M = 0.15 for the girls versus Z-score M= −0.18 for the boys). Clear gender differences in mental health outcomes can be observed in Fig. 1. Compared to boys, girls in general had worse mental health outcomes. Girls had lower self-esteem (M = 19.4 versus M = 20.9), lower subjective well-being (M = 7.5 versus M = 7.8), more depression/anxiety symptoms (M = 7.3 versus M = 3.7) and more perceived stress (M = 19.8 versus M = 15.3) (Supplemental Table 6). Conduct problems were higher in boys than in girls (M = 1.0 versus M = 0.7) as can be seen in Table 1. All differences between boys and girls were statistically significant (*p* < 0.05).

### Association between self-control and mental health

A higher level of self-control was significantly associated with higher self-esteem, higher subjective well-being, fewer depression/anxiety symptoms and less perceived stress in both boys and girls (model 1, Table 2). Gender was a statistically significant effect modifier in the case of self-esteem (*p* = 0.003), subjective well-being (*p* = 0.02) and depression/anxiety symptoms (*p* = 0.03), with effect sizes being larger in girls compared to boys (see also Supplemental Fig. 4). Gender was not a significant effect modifier for perceived stress, with effect sizes being similar in boys and girls (*p* = 0.84). Figure 1 graphically depicts the associations between self-control and the outcome variables for boys and girls. When adjusted for SES (model 2) all the associations remained significant (*p* < 0.01) for both boys and girls, with only small changes in the regression coefficients (Table 2).

**Table 2:** Results of the analysis of the associations between self-control and the mental health outcomes using linear regression stratified by gender

	Model 1		Model 2	
	Univariate		Adjusted for SES	
Outcome	B [95% CI]	*p*-value	B [95% CI]	*p*-value
Self-esteem	*n* = 1505		*n* = 1477	
Girls	1.22 [0.85, 1.59]	< 0.001	1.17 [0.80, 1.55]	< 0.001
Boys	0.48 [0.16, 0.79]	0.003	0.46 [0.13, 0.78]	0.006
Subjective well-being	*n* = 1683		*n* = 1649	
Girls	0.34 [0.21, 0.47]	< 0.001	0.36 [0.23, 0.49]	< 0.001
Boys	0.15 [0.04, 0.27]	0.008	0.16 [0.05, 0.28]	0.005
Depression/anxiety symptoms	*n* = 1773		*n* = 1736	
Girls	−1.07 [−1.55, −0.60]	< 0.001	−1.17 [−1.66, −0.68]	< 0.001
Boys	−0.44 [−0.75, −0.13]	0.005	−0.47 [−0.78, −0.15]	0.004
Perceived stress	*n* = 1434		*n* = 1408	
Girls	−1.23 [−1.88, −0.57]	< 0.001	−1.16 [−1.83, −0.49]	< 0.001
Boys	−1.18 [−1.71, −0.65]	< 0.001	−1.15 [−1.69, −0.62]	< 0.001

*SES *socioeconomic status

### Mediation by conduct problems

Table 3 shows the results of the mediation analysis stratified by gender. Self-control and conduct problems were significantly associated (*p* < 0.001) and showed approximately the same size associations in boys and girls (a-paths). Conduct problems and the four mental health outcomes were not significantly associated in boys (b-paths) and thus the indirect effects in boys were also not statistically significant. In girls, conduct problems were significantly associated with all four outcome variables (b-paths) and indirect effects were all statistically significant with CIs not containing zero. For girls, conduct problems functioned as a partial mediator in the associations between self-control and self-esteem, subjective well-being and depression/anxiety symptoms and as a full mediator in the association between self-control and perceived stress.

**Table 3:** Results of mediation analyses, stratified by gender

	a	b	c (total effect)	c’ (direct effect)	a x b (indirect effect)
Outcome	B [95% CI)	B [95% CI]	B [95% CI]	B [95% CI]	B [95% CI]
Self-esteem Girls Boys	−0.75 [−0.85, −0.66]* −0.64 [−0.72, −0.55]*	−0.39 [−0.65, −0.13]* −0.28 [−0.56, 0.01]	1.22 [0.85, 1.59]* 0.46 [0.13, 0.78]*	0.92 [0.51, 1.34]* 0.28 [−0.09, 0.65]	0.30 [0.08, 0.53]* 0.18 [−0.01, 0.36]]
Subjective well-being Girls Boys	−0.69 [−0.78, −0.60]* −0.69 [−0.77, −0.60]*	−0.16 [−0.26, −0.07]* −0.06 [−0.16, 0.04]	0.34 [0.21, 0.47]* 0.16 [0.05, 0.28]*	0.23 [0.09, 0.37]* 0.12 [−0.01, 0.25]	0.11 [0.05, 0.18]* 0.04 [−0.03, 0.11]
Depression/anxiety symptoms Girls Boys	−0.72 [−0.81, −0.63]* −0.70 [−0.77, −0.62]*	0.63 [0.29, 0.98]* 0.26 [−0.01, 0.54]	−1.07 [−1.55, −0.59]* −0.47 [−0.78, −0.15]*	−0.62 [−1.15, −0.08]* −0.29 [−0.65, 0.08]	−0.45 [−0.75, −0.08]* −0.18 [−0.42, 0.04]
Perceived stress Girls Boys	−0.71 [−0.81, −0.62]* −0.63 [−0.71, −0.54]*	0.51 [0.04, 0.98]* −0.01 [−0.49, 0.47]	−1.20 [−1.86, −0.55]* −1.15 [−1.69, −0.61]*	−0.84 [−1.57, 0.11] −1.15 [−1.77, −0.54]*	−0.37 [−0.73, −0.11]* 0.00 [−0.28, 0.27]

*p < 0.05; a = The association between self-control and conduct problems (controlled for SES)*, b = *The association between conduct problems and mental health outcomes,* c = T*he association between self-control and mental health outcome (controlled for SES),* c’= *The association between self-control and mental health outcomes controlled for conduct problems (and SES),* SES s*ocioeconomic status

### Sensitivity analysis

Models of the sensitivity analysis with IQ included (models 1a and 2a) had notably fewer children included than in our main analyses (*n* = 1211 versus *n* = 1775, Table 4). We observed the same overall trends in the children that had an IQ score available as in our main analysis (model 1a).

**Table 4:** Results of the sensitivity analysis with IQ included for the associations between self-control and the mental health outcomes using linear regression

	Model 1a		Model 2a	
	Univariate model		Adjusted SES and IQ	
Outcome	B [95% CI]	*p*-value	B [95% CI]	*p*-value
Self-esteem	*n* = 1049		*n* = 1038	
Girls	1.03 [0.56, 1.50]	< 0.001	0.99 [0.50, 1.48]	< 0.001
Boys	0.36 [−0.01, 0.73]	0.05	0.32 [−0.07, 0.70]	0.10
Subjective well-being	*n* = 1159		*n* = 1148	
Girls	0.32 [0.16, 0.47]	< 0.001	0.30 [0.14, 0.47]	< 0.001
Boys	0.06 [−0.06, 0.19]	0.31	0.08 [−0.05, 0.21]	0.24
Depression/Anxiety symptoms	*n* = 1211		*n* = 1198	
Girls	−1.04 [−1.65, −0.42]	< 0.001	−1.17 [−1.80, −0.54]	< 0.001
Boys	−0.19 [−0.55, 0.18]	0.32	−0.24 [−0.62, 0.14]	0.21
Perceived stress	*n* = 1010		*n* = 1001	
Girls	−1.20 [−2.02, −0.39]	0.004	−1.09 [−1.93, −0.25]	0.01
Boys	−0.93 [−1.55, −0.31]	0.003	−0.96 [−1.60, −0.31]	0.004

*SES *socioeconomic status

## Discussion

In this study, we investigated the longitudinal association between self-control during childhood from 5 to 12 years of age and mental health in adolescence at 15–16 years of age. To the best of our knowledge, we were the first to test whether this longitudinal association was different for boys and girls and to formally test whether conduct problems mediated the potential link between self-control and subsequent mental health.

We hypothesized that higher self-control during childhood would be associated with better mental health outcomes, including higher self-esteem, higher subjective well-being, fewer depression/anxiety symptoms and less perceived stress in adolescence and our study findings confirmed this hypothesis. This association between childhood self-control and adolescent mental health was independent from the SES of the family of the child and IQ of the child. Our findings add to the limited evidence from longitudinal studies, which has shown associations between self-control during childhood and life satisfaction and anxiety but not depressive symptoms [17, 22, 23]. Inconsistencies between the latter findings and our own finding of an association between self-control and depressive symptoms may be explained by methodological differences between studies. For example, in the measurement of depressive symptoms (continuous measure of depressive symptoms in the current study versus categorical measures in [17]), or due to the difference in age when depressive symptoms were measured (15–16 years in the current study versus 11–14 [23] and 30 years [17] in the other longitudinal studies), or due to differences in follow-up time being long versus very short [23]. On the other hand, a meta-analysis by Robson et al. from 2020 provided evidence that preschool and early school self-regulation, a concept closely related to self-control, was negatively associated with depressive symptoms in later school years and that higher childhood self-regulation was associated with a decreased likelihood of depression in adulthood [39]. This evidence is in line with our findings.

Our study showed clear gender differences in self-control, mental health outcomes and the associations between these variables. Girls had higher self-control than boys, while boys displayed more conduct problems and had better mental health, displaying higher self-esteem and subjective well-being and lower levels of depression/anxiety symptoms and perceived stress. These gender differences are all well known in the literature [5, 6, 40, 41]. In addition to these differences, we also showed that the associations between self-control in childhood and mental health outcomes in adolescence were stronger in girls than in boys. In other words, girls’ mental health seemed to be more affected by their self-control than boys’ mental health. These gender differences were especially pronounced for the associations of self-control and self-esteem, subjective well-being and depression/anxiety symptoms and not so much for the association between self-control and perceived stress. To the best of our knowledge, this gender difference for the association between self-control and mental health has not been tested in previous studies. A potential explanation for this gender divergence may be found in a number of cognitive and social characteristics that have previously been demonstrated to differ between boys and girls. Girls have been shown to display more need for acceptance and success, to be more affected by the opinion of their peers and that they are expected to act more according to social rules than boys [42, 43]. In case of lower levels of self-control, these factors may make girls more vulnerable for adverse mental health outcomes than boys.

When investigating conduct problems as a potential mediator in the association between self-control and mental health, we again came across gender differences. While boys had more conduct problems than girls and there was a strong association between self-control and conduct problems in boys, the mental health of boys was not related to their conduct problems. In girls, however, conduct problems and mental health outcomes were shown to be strongly associated and conduct problems functioned as a mediator in the associations between self-control and mental health outcomes. These findings support other studies that demonstrated a link between childhood conduct problems and later mental health problems, especially when conduct problems are persistent [20, 21]. Exactly why girls seem more affected by conduct problems in terms of their mental health is unclear. As with self-control, conduct problems may be more of a problem for girls due to the potentially stronger need for acceptance, success and approval by their peers and the stronger need to act according to social rules [42, 43]. We do have to note that conduct problems were measured at age 11–12, while our self-control measure was based on measurements at age 5–6 and age 11–12. Our hypothesis that conduct problems may mediate the association between self-control and mental health problems was based on prior evidence suggesting this relationship [17–21]. Although our results suggest such mediation in girls, the cross-sectional timing of part of the self-control measure and the mediator limits causal interpretation.

Our self-control measure was based on items of several questionnaires filled out by multiple reporters, including parents, teachers and the children themselves, at ages 5–6 and 11–12 years. We found that the self-control measure showed weak to moderate stability in our population from the ages 5–6 to 11–12. Other studies that examined the stability of self-control in children found similar stability indices [44–46]. These findings support evidence that despite prior assumptions of self-control theory that self-control skills are resistant to change from about 8–10 years old and despite its genetic components [47, 48], self-control skills can indeed change during adolescence [49]. Indeed, interventions aimed at improving self-control in childhood can be effective [50, 51]. This malleability of self-control in childhood is important as it could provide strategies for prevention of adverse mental health outcomes.

Our study has several strengths and limitations. One of the main strengths is the longitudinal character of our study and its relatively large sample size. Previous studies focusing on self-control and related outcomes (in adolescents and adults) were often cross-sectional and therefore the evidenced associations could very easily be bidirectional. However, in absence of the data, we were unable to correct for baseline levels of mental health at ages 5–6 or 11–12, so it was not possible to conduct more rigorous longitudinal mediation and take into account whether mental health outcomes at younger ages affected self-control. Another strength is that we did not categorize self-control and our outcome variables but kept them continuous for our main analyses, preventing (arbitrary) cut-off points. The use of a multi-occasion (at different ages), multi-informant (mother, teacher, child) approach, following the studies by Moffit et al. [8], Fergusson et al. [17], and Richmond-Rakerd et al. [22], offers several advantages, but also presents certain limitations. Rather than focusing on a specific developmental period, this strategy provides a broader, more comprehensive picture of self-control across childhood. Incorporating multiple informants helps to mitigate potential biases associated with relying on a single source and allows for a more contextually rich assessment across different settings. However, this approach can introduce temporal confounding, since behaviors and their interpretation may change with age, making items assessed at different time points potentially non-equivalent. Also, our mediation analysis partly violated temporality of different variables in the model, as the independent variable as well as the mediator were based on data collected at age 11–12, with the mediator information coinciding with the independent variable information in time. Another limitation lies in the variation across informants. Different raters may interpret questions differently or observe distinct aspects of a child’s behavior, which can complicate the interpretation of what the composite self-control measure truly reflects. A final limitation of our study is that our non-response analysis showed selective loss to follow-up. The included children were more often of higher educated parents with the Dutch ethnicity of maternal origin than the excluded children. This could have caused some selection bias in our study and therefore it is unclear whether these results can be generalized to fit all children with different educational and ethnic backgrounds.

Mental health problems often first manifest during adolescence and are a growing problem worldwide. The present study adds strong evidence that low self-control during childhood is associated with poorer mental health during adolescence and points at lower self-control as a potential precursor of mental health problems. Our findings also show that gender is an important factor when studying self-control effects on mental health, with girls more strongly affected by having low self-control in relation to their mental health, despite boys generally having lower self-control. Moreover, in girls we found evidence that conduct problems may function as a mediator in the pathway between self-control and mental health, while in boys this was not the case.

We also found that self-control showed weak to moderate stability from the ages 5–6 to 11–12, suggesting that this age period could be a window of opportunity for prevention and intervention strategies to enhance self-control, with benefits for mental health in later life.

## Supplementary Information

Below is the link to the electronic supplementary material.

Supplementary Material 1 (DOCX 714 KB)

## Data Availability

The data that support the findings of this study are not openly available due to reasons of sensitivity and are available from the corresponding author upon reasonable request.
