# Supplementary material for: Self-control during childhood and mental health at age 15–16: a longitudinal population-based study
Source: Eur Child Adolesc Psychiatry. 2025 Oct 29;35(3):911–21. doi: 10.1007/s00787-025-02898-0 (PMC13212649; doi:10.1007/s00787-025-02898-0)

*Supplemental Table 1. Self-control items used to establish self-control skills during childhood*

| Questionnaire and its domain |  | Age (years) | Source | Item content |
| --- | --- | --- | --- | --- |
| SDQ (hyperactivity/ inattention domain) |  | 5-6 | Mother, teacher | - Restless or overactive  - Constantly fidgeting or squirming  - Easily distracted and concentration wanders  - Does not thinks things out before acting  - Does not see tasks through to the end, bad attention span |
| SDQ (hyperactivity/ inattention domain) |  | 11-12 | Mother, teacher, child | - Restless or overactive  - Constantly fidgeting or squirming  - Easily distracted and concentration wanders  - Does not thinks things out before acting  - Does not see tasks through to the end, bad attention span |
| BRIEF (working memory domain, emotional control domain and inhibition domain) |  | 11-12 | Mother | - Short attention span - Trouble focusing on jobs or tasks - Needs help from an adult to focus on a task  - Has outbursts for little reasons  - Gets upset over small events  - Talks at the wrong time - Easily overwhelmed  - Acts wild or unruly  - Has trouble putting a brake on their behavior |
| SURPS (impulsivity domain) |  | 11-12 | Child | - Often does not think things through before speaking  - Often involved in situations they later regret being involved in - Acts without stopping to think - Generally are an impulsive person - Feel they have to manipulate to get what they want |

*SDQ = Strengths and Difficulties Questionnaire, BRIEF = Behavior Rating Inventory of Executive Functions, SURPS = Substance Abuse Risk Profile Scale*

*Supplemental Table 2. Non-response analysis.*

|  | Included children | Excluded children | p-value |
| --- | --- | --- | --- |
| **Categorical variables** | | | |
|  | n=1775 (%) | n=2807 (%) |  |
| Ethnicity of maternal origin |  |  |  |
| Dutch | 1370 (77.2) | 1599 (57.0) | <0.001^a^ |
| Other western | 214 (12.1) | 338 (12.0) | 0.99 ^a^ |
| Non-western | 191 (10.8) | 870 (31.0) | <0.001 ^a^ |
| *Missing* | *0 (0)* | *0 (0)* |  |
| Household |  |  |  |
| Two-parent household | 1601 (90.2) | 2240 (79.8) | <0.001^a^ |
| Single-parent household | 107 (6.0) | 337 (12.0) | <0.001^a^ |
| Other | 64 (3.6) | 140 (5.0) | 0.03^a^ |
| *Missing* | *3 (0.2)* | *90 (3.2)* |  |
| Gender |  |  |  |
| Boys | 829 (46.7) | 1471 (52.4) | <0.001^a^ |
| Girls | 946 (53.3) | 1336 (47.6) | <0.001^a^ |
| *Missing* | *0 (0)* | *0 (0)* |  |
| SES parents (highest education) |  |  |  |
| ISCED 1 | 83 (4.7) | 559 (19.9) | <0.001^a^ |
| ISCED 2 | 307 (17.3) | 630 (22.4) | <0.001^a^ |
| ISCED 3 | 1372 (77.3) | 1474 (52.5) | <0.001^a^ |
| *Missing* | *13 (0.7)* | *144 (5.1)* |  |
| **Continuous variables** | | | |
|  | Mean (SD) | Mean (SD) | p-value |
| Age |  |  |  |
| Age mother phase 3 (years) | 38.1 (4.4) | 36.8 (5.9) | <0.001^b^ |
| *Missing n(%)* | *77 (4.4)* | *485 (17.3)* |  |
| Age father phase 4 (years) | 40.6 (5.5) | 39.9 (6.0) | <0.001^b^ |
| *Missing n(%)* | *156 (8.8)* | *670 (23.9)* |  |
| Conduct problems |  |  |  |
| SDQ conduct problems (score) | 0.8 (0.7) | 1.1 (1.1) | <0.001^b^ |
| *Missing n(%)* | *0 (0)* | *17 (0.6)* |  |

*^a^ Pearson Chi-square, ^b^ T-test
SES= Socioeconomic status, ISCED= International Standard Classification of Education, SDQ= Strengths and Difficulties Questionnaire.*

*Supplemental Table 3. Summary statistics for the conduct problems and self-control scales per age group according to gender.*

|  | Age 5-6 years | | | Age 11-12 years | | |
| --- | --- | --- | --- | --- | --- | --- |
|  | Boys | Girls | All | Boys | Girls | All |
| SDQ conduct problems, mother | 1.0 (1.2) | 0.8 (1.1) | 0.9 (1.2) | 0.9 (1.2) | 0.6 (0.9) | 0.7 (1.1) |
| SDQ conduct problems, teacher | 0.8 (1.3) | 0.5 (1.0) | 0.7 (1.1) | 1.4 (1.3) | 1.2 (1.1) | 1.3 (1.2) |
| SDQ conduct problems, child |  |  |  | 0.6 (1.0) | 0.3 (0.7) | 0.4 (0.9) |
| SDQ hyperactivity, mother | 2.4 (2.1) | 1.8 (1.9) | 2.1 (2.0) | 3.1 (2.5) | 2.0 (2.2) | 2.5 (2.4) |
| SDQ hyperactivity, teacher | 2.5 (2.5) | 1.4 (2.1) | 1.9 (2.4) | 2.5 (2.7) | 1.2 (1.8) | 1.8 (2.4) |
| SDQ hyperactivity, child |  |  |  | 3.6 (2.4) | 3.1 (2.3) | 3.3 (2.3) |
| SURPS, child |  |  |  | 9.5 (3.1) | 9.2 (3.0) | 9.4 (3.0) |
| BRIEF working memory + emotional control +inhibition, mother |  |  |  | 13.5 (3.4) | 12.3 (3.1) | 12.9 (3.3) |

*Data are givens as means (SD),* *SDQ = Strengths and Difficulties Questionnaire, BRIEF = Behavior Rating Inventory of Executive Functions, SURPS = Substance Abuse Risk Profile Scale*

*Supplemental Table 4. The development/stability of self-control from age 5-6 (phase 3) to age 11-12 (phase 4)*

|  |  | Self-control in quintiles at age 11-12 | | | | | |
| --- | --- | --- | --- | --- | --- | --- | --- |
|  |  | Q1 (4) | Q2 (4) | Q3 (4) | Q4 (4) | Q5 (4) |  |
|  |  | n(%) | n(%) | n(%) | n(%) | n(%) |  |
| Self-control in quintiles at age 5-6 | Q1 (3) | 163 (9.2) | 85 (4.8) | 52 (2.9) | 40 (2.3) | 20 (1.1) | 360 |
|  | Q2 (3) | 83 (4.7) | 85 (4.8) | 73 (4.1) | 56 (3.2) | 41 (2.3) | 338 |
|  | Q3 (3) | 50 (2.8) | 79 (4.5) | 102 (5.7) | 86 (4.8) | 89 (5.0) | 406 |
|  | Q4 (3) | 41 (2.3) | 60 (3.4) | 73 (4.1) | 79 (4.5) | 69 (3.9) | 322 |
|  | Q5 (3) | 18 (1.0) | 46 (2.6) | 55 (3.1) | 94 (5.3) | 136 (7.7) | 349 |
|  |  | 355 | 355 | 355 | 355 | 355 | 1775 |

See also Supplemental figure 3.

*Supplemental Table 5. Correlation table of self-control questionnaires, self-control per age group and overall self-control, stratified per gender.*

|  | 1. | 2. | 3. | 4. | 5. | 6. | 7. | 8. | 9 | 10. | 11. | 12 |
| --- | --- | --- | --- | --- | --- | --- | --- | --- | --- | --- | --- | --- |
| 1. SDQ hyperactivity, mother (age 5-6) | 1 | 0.40** | 0.49 ** | 0.28** | 0.31** | 0.28** | 0.36** | 0.87** | 0.45** | 0.67** | 0.35** | 0.27** |
| 2. SDQ hyperactivity, teacher (age 5-6) | 0.38** | 1 | 0.35** | 0.33** | 0.26** | 0.20** | 0.31** | 0.83** | 0.34** | 0.59** | 0.36** | 0.17** |
| 3. SDQ hyperactivity, mother (age 11-12) | 0.49** | 0.31** | 1 | 0.47** | 0.59** | 0.38** | 0.70** | 0.50** | 0.83** | 0.83** | 0.23** | 0.31** |
| 4. SDQ hyperactivity, teacher (age 11-12) | 0.31** | 0.43** | 0.57** | 1 | 0.44** | 0.27** | 0.38** | 0.35** | 0.47** | 0.64** | 0.20** | 0.33** |
| 5. SDQ hyperactivity, child (age 11-12) | 0.27** | 0.24** | 0.58** | 0.50** | 1 | 0.54** | 0.48** | 0.33** | 0.82** | 0.75** | 0.18** | 0.36** |
| 6. SURPS, child (age 11-12) | 0.21** | 0.12** | 0.38** | 0.29** | 0.58** | 1 | 0.36** | 0.28** | 0.73** | 0.65** | 0.20** | 0.41** |
| 7. BRIEF working memory + emotional control +inhibition, mother (age 11-12) | 0.41** | 0.22** | 0.73** | 0.47** | 0.49** | 0.36** | 1 | 0.40** | 0.80** | 0.75** | 0.30** | 0.47** |
| 8. Self-control (age 5-6) | 0.85** | 0.83** | 0.47** | 0.42** | 0.29** | 0.19** | 0.37** | 1 | 0.47** | 0.73** | 0.41** | 0.27** |
| 9. Self-control (age 11-12) | 0.42** | 0.27** | 0.84** | 0.57** | 0.83** | 0.72** | 0.82** | 0.41** | 1 | 0.93** | 0.27** | 0.48** |
| 10. Total self-control | 0.63** | 0.56** | 0.84** | 0.74** | 0.75** | 0.61** | 0.75** | 0.70** | 0.91** | 1 | 0.37** | 0.48** |
| 11. Conduct problems (age 5-6) | 0.34** | 0.36** | 0.25** | 0.22** | 0.15** | 0.17** | 0.30** | 0.40** | 0.27** | 0.37** | 1 | 0.31** |
| 12. Conduct problems (age 11-12) | 0.28** | 0.11** | 0.41** | 0.40** | 0.42** | 0.43** | 0.54** | 0.24** | 0.56** | 0.52** | 0.35** | 1 |

* Correlation is significant with p<0.05
** Correlation is significant with p<0.01
 = Girls = Boys

*Supplemental Table 6. Self-control in quintiles and the mental health outcome variables, total and stratified by gender.*

|  | Self-control in quintiles | | | | | | | Total |
| --- | --- | --- | --- | --- | --- | --- | --- | --- |
|  | Q1 (lowest) | Q2 | | Q3 | | Q4 | Q5 |  |
| **Outcome variables** | | | | | | | | |
|  | Mean (SD) | | Mean (SD) | | Mean (SD) | Mean (SD) | Mean(SD) | Mean(SD) |
| Self-esteem (score) |  | |  | |  |  |  |  |
| Total | 19.4 (3.7) | | 20.0 (3.5) | | 19.9 (3.4) | 20.2 (3.2) | 20.6 (3.0) | 20.0 (3.4) |
| Boys | 20.4 (3.2) | | 20.9 (2.8) | | 21.0 (3.0) | 21.0 (3.0) | 21.4 (3.1) | 20.9 (3.0) |
| *n* | *176* | | *168* | | *120* | *126* | *88* | *678* |
| Girls | 17.9 (3.9) | | 19.0 (4.0) | | 19.2 (3.4) | 19.7 (3.3) | 20.3 (2.9) | 19.4 (3.5) |
| *n* | *105* | | *138* | | *175* | *188* | *221* | *827* |
| *Missing n(%)* | *74 (20.8)* | | *49 (13.8)* | | *60 (16.9)* | *41 (11.5)* | *46 (13.0)* | *270 (15.2)* |
| Subjective well-being (score) |  | |  | |  |  |  |  |
| Total | 7.5 (1.4) | | 7.5 (1.3) | | 7.6 (1.2) | 7.7 (1.1) | 7.8 (1.1) | 7.6 (1.2) |
| Boys | 7.8 (1.3) | | 7.7 (1.1) | | 7.9 (1.1) | 7.8 (1.0) | 8.1 (1.0) | 7.8 (1.1) |
| *n* | *204* | | *184* | | *144* | *140* | *101* | *773* |
| Girls | 7.1 (1.4) | | 7.3 (1.5) | | 7.5 (1.2) | 7.6 (1.2) | 7.7 (1.1) | 7.5 (1.3) |
| *n* | *120* | | *146* | | *193* | *205* | *246* | *910* |
| *Missing n(%)* | *31 (8.7)* | | *25 (7.0)* | | *18 (5.1)* | *10 (2.8)* | *8 (2.3)* | *92 (5.2)* |
| Depression/  anxiety symptoms (score) |  | |  | |  |  |  |  |
| Total | 5.7 (4.9) | | 5.5 (4.7) | | 5.7 (4.5) | 5.7 (4.7) | 5.2 (4.0) | 5.6 (4.6) |
| Boys | 4.2 (3.9) | | 3.5 (3.0) | | 3.5 (3.1) | 3.6 (3.5) | 3.0 (2.6) | 3.7 (3.3) |
| *n* | *224* | | *198* | | *157* | *146* | *103* | *828* |
| Girls | 8.3 (5.5) | | 8.0 (5.3) | | 7.5 (4.7) | 7.2 (4.8) | 6.1 (4.2) | 7.3 (4.9) |
| *n* | *131* | | *156* | | *198* | *208* | *252* | *945* |
| *Missing n(%)* | *0 (0)* | | *1 (0.3)* | | *0 (0)* | *1 (0)* | *0 (0)* | *2 (0.1)* |
| Perceived stress (score) |  | |  | |  |  |  |  |
| Total | 18.2 (5.9) | | 17.7 (6.2) | | 18.1 (5.8) | 17.8 (5.8) | 17.2 (5.9) | 17.8 (5.9) |
| Boys | 16.6 (5.0) | | 15.2 (5.0) | | 15.1 (4.7) | 15.1 (4.8) | 13.6 (5.0) | 15.3 (5.0) |
| *n* | *165* | | *158* | | *110* | *120* | *87* | *640* |
| Girls | 21.1 (6.2) | | 20.7 (6.2) | | 20.1 (5.7) | 19.6 (5.8) | 18.7 (5.6) | 19.8 (5.9) |
| *n* | *95* | | *129* | | *171* | *185* | *214* | *794* |
| *Missing n(%)* | *95 (26.8)* | | *68 (19.2)* | | *74 (20.8)* | *50 (14.1)* | *54 (15.2)* | *341 (19.2)* |

*Supplemental Figure 1. Flowchart of inclusion to the study.*

n=4582
Children that started phase 3

n=2584

n=1775
Total children included

n=1998
Excluded based on <4 self-control questionnaires or no data self-control phase 3 or 4

n=809
Excluded based on no data for outcome variables available

*Supplemental Figure 2. Overview pathways of models without and with mediation by conduct problems. (a, b, c and c’-path).*


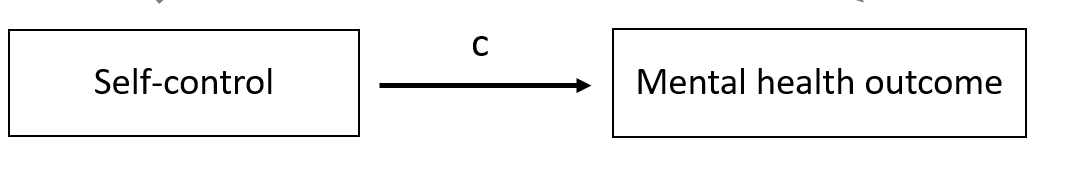


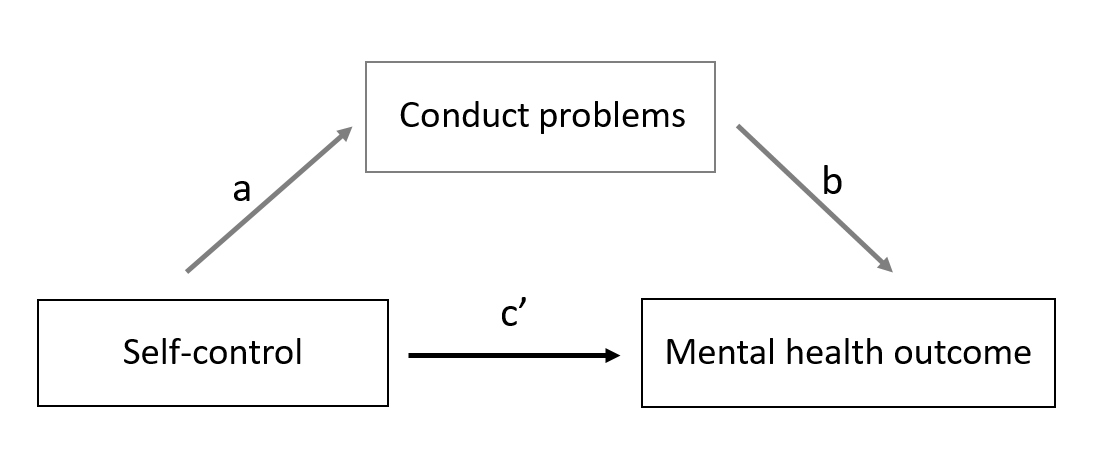


*Supplemental Figure 3. Sankey diagram of the development and stability of self-control in quintiles from age 5-6 years (phase 3) on the left to age 11-12 years (phase 4) on the right.*

*
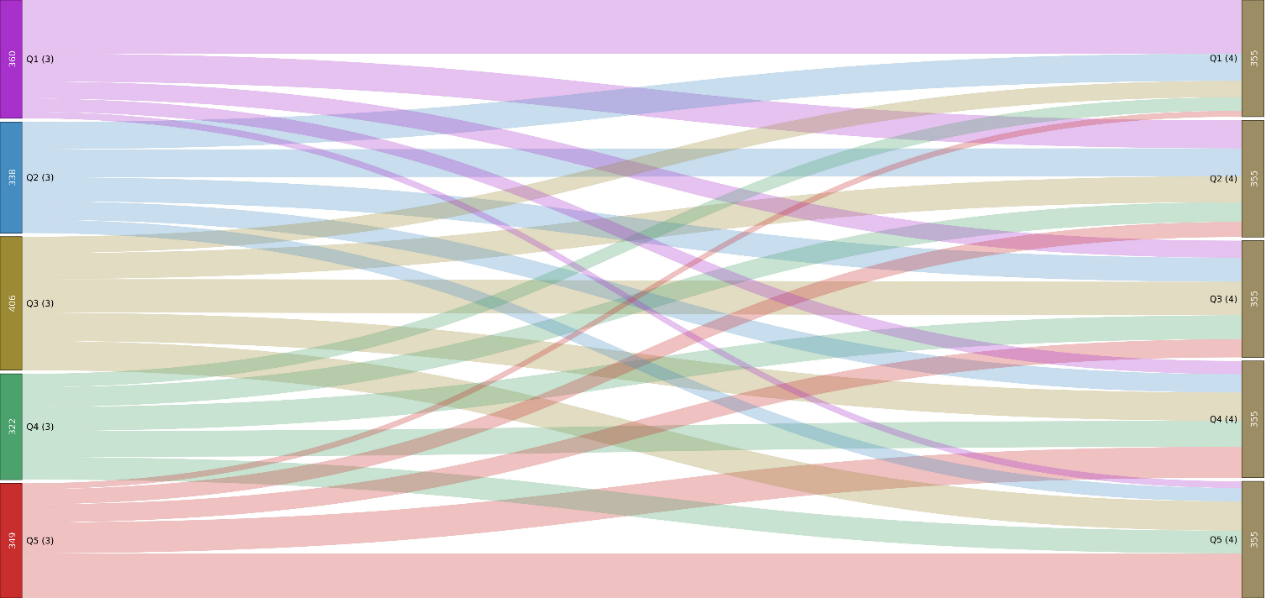
*made by The Sankey Diagram Generator (http://sankey-diagram-generator.acquireprocure.com/)*

*Supplemental Figure 4a. The association between self-control and self-esteem was stronger (steeper slope) for girls than for boys.*


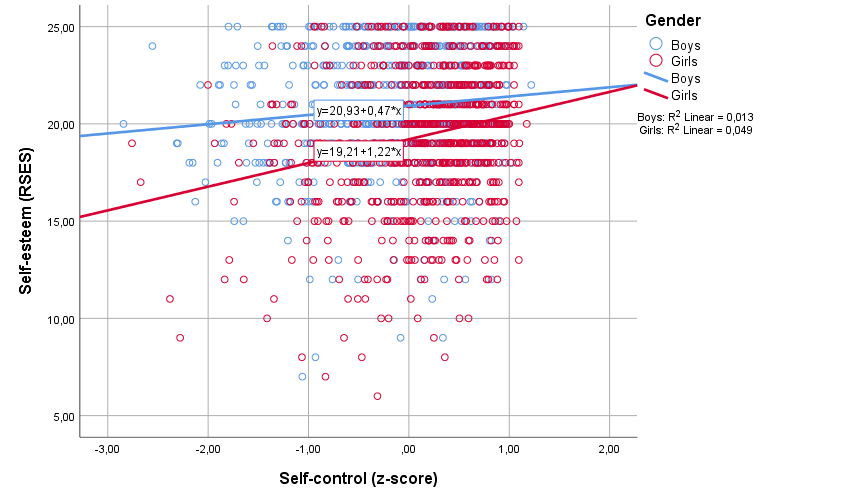


*Supplemental Figure 4b. The association between self-control and subjective well-being was stronger (steeper slope) for girls than for boys.*


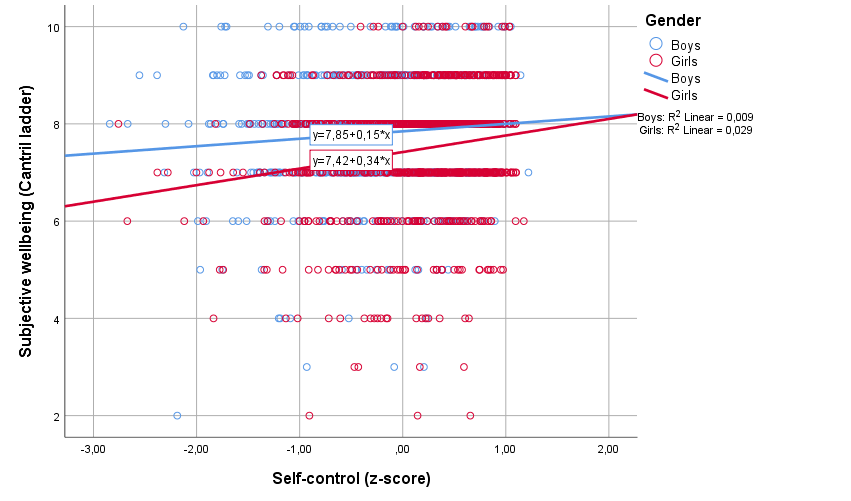


*Supplemental Figure 4c. The association between self-control and depression/anxiety symptoms was stronger (steeper slope) for girls than for boys.*


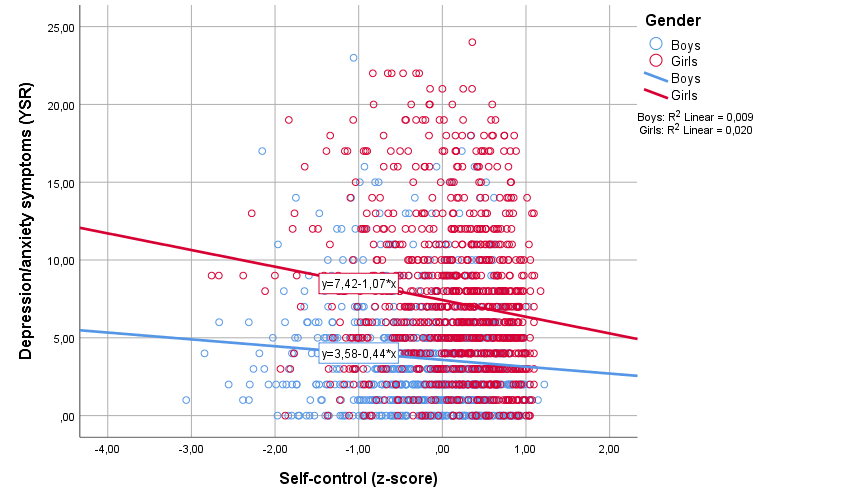


*Supplemental Figure 4d: Gender is not a significant effect-modifier in the association between self-control and perceived stress, the slopes are approximately parallel. The scatterplot does show a different distribution for boys and girls, with girls scoring overall higher on perceived stress than boys.*


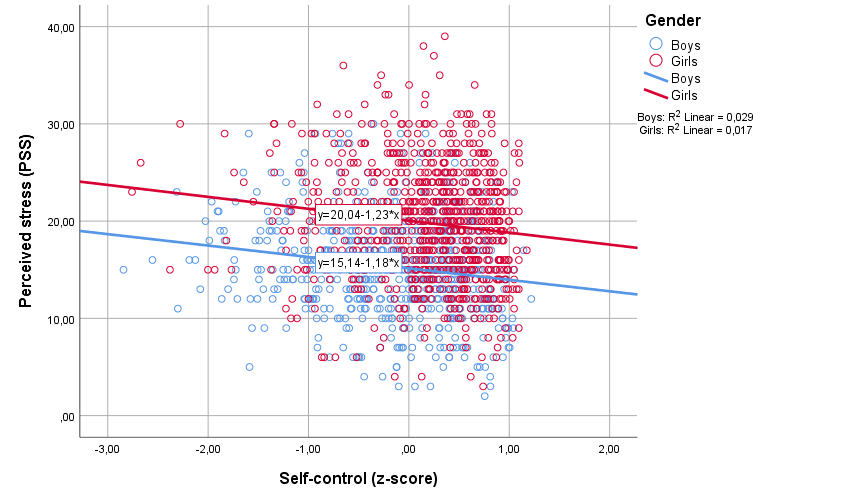

Supplement: Supplementary file 1 — Supplementary Material 1 (DOCX 714 KB) [file 787_2025_2898_MOESM1_ESM.docx]
